# Improved in vitro angiogenic behavior on anodized titanium dioxide nanotubes

**DOI:** 10.1186/s12951-017-0247-8

**Published:** 2017-01-31

**Authors:** Ernesto Beltrán-Partida, Benjamín Valdéz-Salas, Aldo Moreno-Ulloa, Alan Escamilla, Mario A. Curiel, Raúl Rosales-Ibáñez, Francisco Villarreal, David M. Bastidas, José M. Bastidas

**Affiliations:** 1 Department of Biomaterials and Tissue Engineering, Faculty of Dentistry Mexicali, Autonomous University of Baja California (UABC), Ave. Zotoluca and Chinampas St., 21040 Mexicali, Baja California Mexico; 2 Department of Corrosion and Materials, Engineering Institute, Autonomous University of Baja California (UABC), Blvd. Benito Juarez and Normal St., 21280 Mexicali, Baja California Mexico; 3 Department of Biomedical Innovation, Center for Scientific Research and Higher Education of Ensenada (CICESE), Ensenada, Baja California Mexico; 4 Laboratory of Basic Sciences, Faculty of Stomatology, Autonomous University of San Luis Potosi (UASLP), San Luis Potosí, Mexico; 5 School of Medicine, University of California San Diego (UCSD), 9500 Gilman Dr, La Jolla, CA 92093 USA; 6 National Centre for Metallurgical Research (CENIM), CSIC, Ave. Gregorio del Amo 8, 28040 Madrid, Spain

**Keywords:** Endothelialization, Titanium, Nanotubes, Anodization, Super oxidized water, Dental implants

## Abstract

**Background:**

Neovascularization over dental implants is an imperative requisite to achieve successful osseointegration onto implanted materials. The aim of this study was to investigate the effects on in vitro angiogenesis of anodized 70 nm diameter TiO_2_ nanotubes (NTs) on Ti6Al4V alloy synthesized and disinfected by means of a novel, facile, antibacterial and cost-effective method using super oxidized water (SOW). We also evaluated the role of the surface roughness and chemical composition of materials of materials on angiogenesis.

**Methods:**

The Ti6Al4V alloy and a commercially pure Ti were anodized using a solution constituted by SOW and fluoride as electrolyte. An acid-etched Ti6Al4V was evaluated to compare the effect of micro-surface roughness. Mirror-polished materials were used as control. Morphology, roughness, chemistry and wettability were assessed by field emission scanning electron microscopy (FE-SEM), transmission electron microscopy, atomic force microscopy, energy dispersive X-ray spectroscopy (EDX) and using a professional digital camera. Bovine coronary artery endothelial cells (BCAECs) were seeded over the experimental surfaces for several incubation times. Cellular adhesion, proliferation and monolayer formation were evaluated by means of SEM. BCAEC viability, actin stress fibers and vinculin cellular organization, as well as the angiogenic receptors vascular endothelial growth factor 2 (VEGFR2) and endothelial nitric oxide synthase (eNOS) were measured using fluorescence microscopy.

**Results:**

The anodization process significantly increased the roughness, wettability and thickness of the oxidized coating. EDX analysis demonstrated an increased oxygen (O) and decreased carbon (C) content on the NTs of both materials. Endothelial behavior was solidly supported and improved by the NTs (without significant differences between Ti and alloy), showing that endothelial viability, adhesion, proliferation, actin arrangement with vinculin expression and monolayer development were evidently stimulated on the nanostructured surface, also leading to increased activation of VEGFR2 and eNOS on Ti6Al4V-NTs compared to the control Ti6Al4V alloy. Although the rougher alloy promoted BCAECs viability and proliferation, filopodia formation was poor.

**Conclusion:**

The in vitro results suggest that 70 nm diameter NTs manufactured by anodization and cleaned using SOW promotes in vitro endothelial activity, which may improve in vivo angiogenesis supporting a faster clinical osseointegration process.

## Background

Titanium (Ti) and Ti-based alloys (i.e. Ti6Al4V) are the most widely used materials for dental implants. However, problems such as mechanical and biocompatibility issues associated with long-term use continue to lead to incomplete success and clinical failure. From a clinical point of view rapid osseointegration around the implanted material [1–4] is an important pre-requisite for successful tissue regeneration throughout the implant, the prevention of early implant-associated infection (e.g. peri-implant mucositis and peri-implantitis) [5, 6], and most importantly to achieve a prompt and durable effective clinical result. Furthermore, as bone is a highly vascularized tissue, another requirement for proper osseointegration is the correct stimulation of angiogenesis by the material’s surface. Angiogenesis is defined as the process of new blood vessel formation by endothelial cells, strongly supporting new bone formation by delivering nutrients, regulatory factors i.e. vascular endothelial growth factors (VEGF) and nitric oxide (NO) synthesized by the phosphorylation of endothelial nitric oxide synthase (e-NOS), oxygen and a numerous cell types including inflammatory and osteoprogenitor cells in implanted materials [7–9].

It has been suggested that the physicochemical properties of implant material surfaces, such as roughness, wettability, chemistry and surface morphology, may play a pivotal role in the control of cellular and clinical responses to implant treatments such as osteoblast maturation and endothelial remodeling. Plasma-spraying has been applied on Ti-based implants to produce rougher surfaces, but may lead to the generation and migration of Ti wear particles into adjacent blood vessels and bone, in the liver, spleen, small aggregates of macrophages and even in the para-aortic lymph nodes [10], information that must be taken as a concern by the clinician. Surface modification can also be achieved by grit-blasting using hard ceramic particles [11], but this physical method often involves the use of blasting materials like alumina (Al_2_O_3_), and a number of clinical reports have indicated the presence of Al_2_O_3_ particles in the surrounding tissues, impeding the osseointegration of implants probably due to chemical interferences between the adjacent tissues and the alloy surfaces [11]. Another commonly used surface modification technique is the immersion of implant screws in concentrated and heated acid mixtures [11], though this method may regrettably be detrimental to the strict demands of mechanical performance of Ti-based implants [12], mainly due to the dissolution process that takes place on the surface of the material.

Nanostructured materials offer promising strategies for the promotion of improved cell growth, tissue remodeling and even angiogenesis [13]. For instance, anodization is an electrochemical method that allows the simple formation of self-organized, ordered and well-aligned NTs across the metal surface [14–17]. Anodizing increases the surface area, improves corrosion resistance, raises surface roughness, promotes a smaller water contact angle, and most significantly improves biocompatibility compared to non-modified surfaces [18–21]. Importantly, SOW is a neutral-pH antibacterial electrolyzed water that is mainly composed by oxidizing radicals (i.e. hypochlorous species), H_2_O_2_ and chlorine molecules [22]. SOW solution is widely accepted as an efficient disinfectant; due to its potent antibacterial/antimycotic capability without altering the microstructural (non-corrosive) and mechanical properties of metallic medical materials [23]. For instance, the use of SOW as a disinfectant has been successfully used for wound healing procedures due to its capability for not stimulate an inflammatory reaction without interfering in the angiogenesis required for tissue regeneration [24]. Furthermore, in a recent study the authors reported the beneficial effects of SOW for performing faster and more simple NT synthesis compared to other published methods and for the disinfection of NT surfaces [25]. Using this protocol led to a significant decrease in *Staphylococcus aureus* (*S. aureus*) adhesion and viability without negatively altering osteoblast and chondrocyte behavior [14, 25]. Interestingly, *S. aureus* is an anaerobic facultative gram-positive bacteria that is emerging as an important pathogen associated with the etiology of early stages of the peri-implantitis process [5, 26], contributing to the formation of deep peri-implant pockets and also strongly associated with suppuration and bleeding on probing [5, 27, 28]. There is information which suggests that NTs synthesized and cleaned with SOW may decrease the adhesion ability of important periodontal pathogens (such as *S. aureus*) without affecting the biocompatibility of the NT surface, but the vital in vitro endothelialization process on these NT surfaces has not yet been elucidated.

Thus, considering the above-stated information and given the importance of angiogenesis in stimulating osteogenesis, the authors wish to test for the first time the hypothesis that Ti6Al4V alloy configured with NTs synthesized and cleaned using SOW enhances in vitro angiogenesis compared to a flat non-modified Ti6Al4V alloy. This study evaluates the angiogenesis process of BCAECs by means of cellular adhesion, proliferation, vinculin stimulation and formation of an endothelial monolayer. In addition, we also evaluate the role of the surface roughness and chemical composition of Ti-based materials on angiogenesis. We have also investigated the activation of angiogenic factors such as e-NOS and VEGFR2 receptors that are largely required for the promotion of angiogenesis.

## Methods

### Synthesis of NTs

The synthesis and cleaning process for NTs was performed as described previously [25]. In brief, Ti6Al4V discs (ASTMF-136, Supra Alloys Inc, Camarillo, CA, USA) of 150 mm diameter and 5 mm thickness were polished using SiC emery paper (100–2000 grit) and 1-μm alumina to achieve a mirror finish. Next, the samples were mounted on a special flat 125 mL cell and electrolytically anodized using Microdacyn 60^®^ SOW (Oculus technologies, Guadalajara, JAL, MEX) at pH 6.8, enriched with 10 mg/L NH_4_F (Sigma Aldrich, USA) and 100 mg/L NaCl (Sigma-Aldrich, USA). A 20 V potential was applied using a DC power supply for 5 min and a platinum mesh as a counter electrode. The process was carried out at room temperature (described here as RT). Lastly, the anodized samples were cleaned in an ultrasonic bath with distilled water for 5 min to eliminate residues of fluorite salts, rinsed with isopropyl alcohol, and dried in a desiccator for 12 h. To achieve a cleaned and disinfected surface, the anodized materials were submerged in 20 mL SOW for 1 h and finally dried at RT before use. A non-anodized and mirror finished Ti6Al4V alloy disc used as control. In order to test the effect of elemental composition in the endothelial morphology and vitality, we included a cp-Ti (grade 4, Supra Alloys Inc, Camarillo, Ca, USA) that was mirror polished and anodized by SOW as described above. Additionally, a non-anodized cp-Ti was included as control. All experimental materials were sterilized by UV irradiation (285 nm UVB light source) for 30 min each side.

### Rough Ti6Al4V by acid-etching

Ti6Al4V discs of 150 mm diameter and 5 mm thickness were polished to a mirror finish. In order to significantly increase the surface roughness of the alloy discs; the materials were immersed in an aqueous solution of 18% HCl and 49% H_2_SO_4_ for 40 min at 60 °C [29]. Then, discs were ultrasonically cleaned in pure water for 15 min, dried in a desiccator for 12 h and sterilized by UV irradiation for 30 min each side.

### Surface characterization

The surface and cross-sectional morphology of the experimental samples (labeled as Ti6Al4V-NTs, Ti6Al4V alloy, cp-Ti, cp-Ti-NTs and Ti6Al4V-rough) were characterized by FE-SEM (Tescan LYRA 3, Brno, Czech Republic), taking images at 20 kV accelerating voltage and the tubes diameter where measured using the top-view analyses. The cross-section of the NTs was analyzed by breaking a 1 cm^2^ anodized foil [30]. Moreover, TEM (using a Jeol 2010 transmission electron microscope operating at a voltage of 200 kV) was used as an analysis to corroborate the surface morphology and the diameter of the Ti6Al4V-NTs. The chemical constitution of the material surfaces was assessed by EDX (Tescan LYRA 3, Brno, Czech Republic) using a silicon drift detector coupled to the FE-SEM.

The topological cues and surface roughness of the experimental materials were evaluated by AFM (Quesant Q-Scope 350, AMBIOS, Agura Hills, CA, USA) analysis at RT. Additionally, the AFM examination was carried out using an anti-acoustic box to prevent noise which can affect the measurements. Topographic images were obtained operating at a scan rate of 1 Hz. A 40-µm X–Y and 4-µm Z scanner equipped with silicon tips and 10 nm tip curvature was used. The experiment scan surface was 4 µm^2^. With the purpose of analytically comparing the surface roughness of the materials, the root mean square (RMS) was calculated and is provided.

In order to evaluate the surface wettability of the experimental materials (Ti6Al4V alloy and Ti6Al4V-NTs), the static sessile-droop method was conducted at RT to obtain the water contact angle following the procedure reported elsewhere [31, 32]. A 15 µL droplet of double-distilled water was dripped onto each material surface using the tip of a syringe, and the shape of the droplet was evaluated taking images using a professional digital camera (Nikon, 7200, NY, USA). The contact angles were analyzed by means of magnified images and the analytical quantification was assessed using Image J software (1.48v, NIH, USA).

### BCAEC culture

BCAECs were purchased from Cell Applications, Inc. (San Diego, CA, USA). The cells were maintained at 37 °C in an incubator with a humidified atmosphere of 5% CO_2_ and cultured in low glucose (1 g/l) Dulbecco’s Modified Eagle’s Medium (DMEM) (Invitrogen, Carlsbad, CA, USA) supplemented with 10% fetal bovine serum (FBS) (Invitrogen, Carlsbad, CA, USA) and 1% antibiotic and antimitotic solution (Invitrogen, Carlsbad, CA, USA). NTs, Ti6Al4V-rough and the control discs were placed in individual wells of a 12-well polystyrene tissue culture plate. The cells were seeded at a cell density of 1 × 10^4^ cells per mL onto the materials with 1 mL of cell media and incubated for different experimental times.

### Viability and cellular proliferation

In order to evaluate the proliferation rate and the viability of BCAECs on the material surfaces, a LIVE/DEAD Viability/Cytotoxicity Assay (Invitrogen, Carlsbad, CA, USA) was performed following the instruction provided by the manufacturer at 1, 3 and 8 days of culture. In brief, the cells were washed three times with 1X phosphate buffered saline (PBS) and then stained for 45 min at 37 °C using 1 mM calcein AM, (which acts by measuring intracellular esterase activity) and 2 mg/mL ethidium homodimer-1 (which enters through damaged cell membranes) [14]. The specimens were then inverted onto coverslips with a fluorescence mounting medium (DAKO, Agilent Technologies, Carpinteria, CA, USA), visualized and photographed using a green (live) and red (dead) filter under a fluorescence microscope using similar magnifications (Axio Observer A1, Carl Zeiss, Thornwood, NY, USA).

### Immunofluorescence staining

As stated above, BCAECs were seeded onto the specimens (Ti6Al4V alloy and Ti6Al4V-NTs). Cellular actin stress fibers were evaluated after 1 and 3 days of incubation. In addition, convergence of Vinculin with F-actin filaments was assessed at 1 day of culture on the alloyed surfaces. Additionally, the activity of two key proteins involved in angiogenesis, eNOS and VEGFR2, was analyzed and the phosphorylation levels of their activation residues was measured by immunofluorescence staining for 1, 3 and 8 days of culture. After completion of the specific incubation time, each sample was initially washed three times with warm PBS and fixed with 4% paraformaldehyde for 30 min at RT. Once fixed, they were again washed three times with PBS and permeabilized using 0.1% Triton X-100 in PBS for 20 min. The samples were next washed three times and incubated for 1 h at RT in bovine serum albumin (BSA) blocking solution (1% BSA/1 × PBS) and washed with PBS. Finally the cells were incubated with Alexa Fluor 488 phalloidin, 1:100 dilution (Invitrogen, Carlsbad, CA, USA), in blocking solution for 1 h in order to analyze actin stress fibers [15, 25]. For the analysis of endothelial-phosphorylated proteins and anti-Vinculin the cells were incubated for 2 h in the primary antibodies p-eNOS, p-VEGFR2 and Vinculin, 1:100 dilution (Abcam, Cambridge, MA, USA) in blocking solution at RT. After this, Alexa Fluor 488-labeled anti-rabbit, 1:1000 dilution (Invitrogen, Carlsbad, CA, USA), and Alexa Fluor 594 labeled anti-mouse, 1:1000 dilution (Invitrogen, Carlsbad, CA, USA), each in blocking solution, were used as secondary antibodies for p-eNOS and p-VEGFR2 and Vinculin respectively and incubated for 1 h at RT and washed three times with PBS [33]. Thereafter, the cell nuclei were counter-stained using 4′,6′-diamidino-2-phenylindole (DAPI) (Molecular Probes, Carlsbad, CA, USA) in PBS, incubated for 5 min at RT, and washed three times with PBS [14]. Finally, the specimens were inverted onto coverslips with a fluorescence mounting medium, examined and photographed using a green (actin and p-eNOS), red (p-VEGFR2 and Vinculin) and blue (DAPI) filter by means of a fluorescence microscope under similar magnifications. To measure the fluorescence intensity of p-eNOS and p-VEGFR2, 5-10 micrographs of each sample were taken at each culture point using the same exposure time. The average intensity was measured using Image J software from five random cells on each surface [34].

### BCAEC characterization by SEM

To evaluate morphological changes on BCAECs seeded on the experimental materials, FE-SEM analysis was carried out as described by others [25, 35]. After 4 h, 1, 3 and 8 days of seeding, cells grown on the experimental specimens were washed twice with PBS and fixed with 5% w/v glutaraldehyde (Sigma-Aldrich, St. Louis, MO, USA) in PBS for 2 h. After fixation cells were washed three times with PBS (10 min each). Next, the cells were dehydrated in grade series of ethanol (50, 70, 90 and 100%) for 30 min at each concentration. Finally the samples were sputter-coated with gold (10 nm gold layer) for 8 s and observed at 5 kV accelerating voltage.

### Statistical analysis

At least three independent experiments were performed, each in triplicate. For the FE-SEM and TEM analysis at least five random fields were analyzed for each experimental group. For the quantification of fluorescence intensity, five random cells were selected from five different surfaces. Numerical data were analyzed using GraphPad Prism 6 (GraphPad Software Inc., La Jolla, CA, USA). The significance of differences between group means was determined using two-tailed unpaired Student’s *t* test and one-way ANOVA followed by Tukey’s multiple comparisons test when appropriate. A *P* < 0.05 was considered statistically significant.

## Results

### Surface analysis

**Fig. 1:**
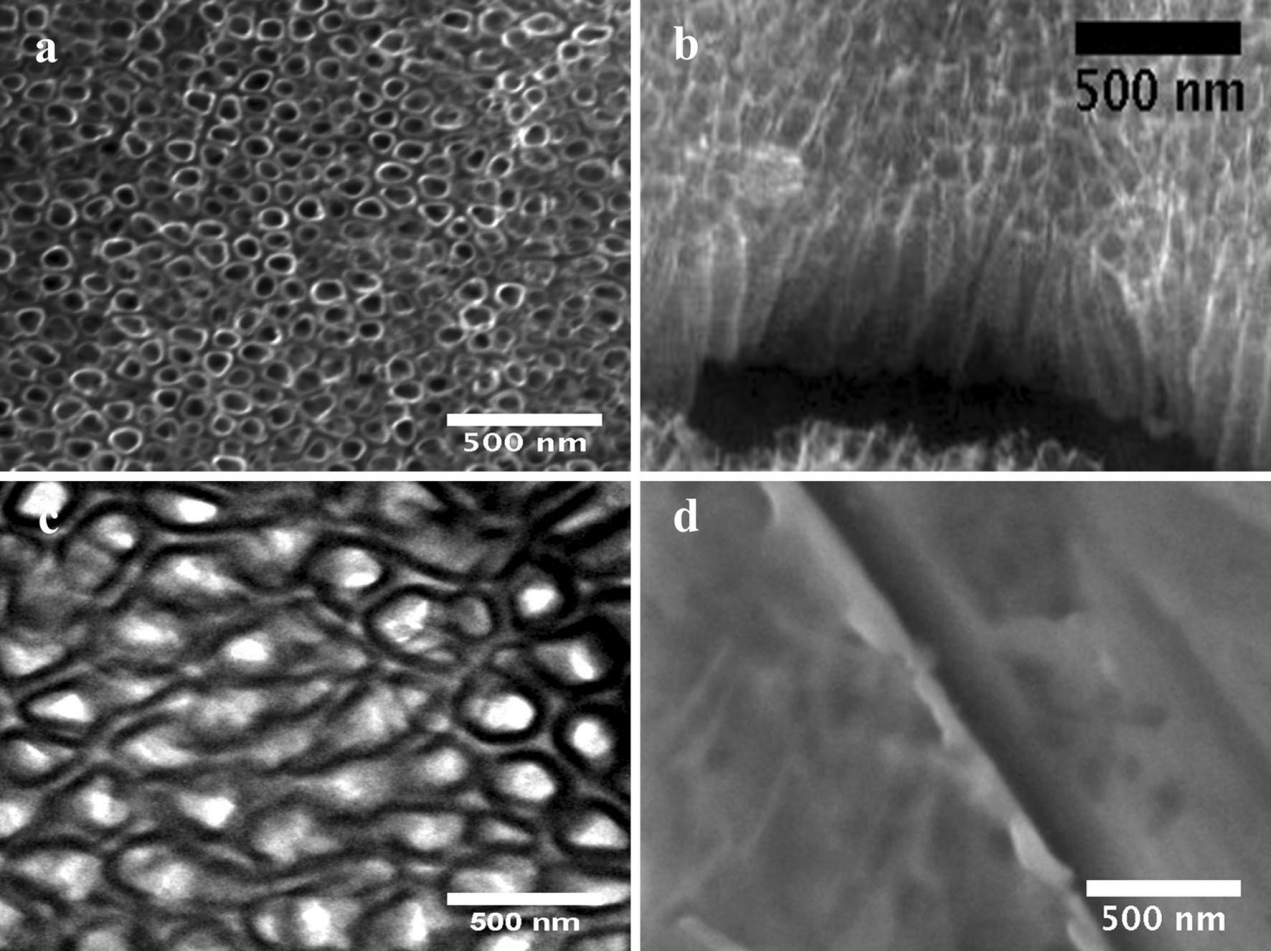
SEM Micrographs representing the surface morphology of the experimental materials. **a** Anodized Ti6Al4V illustrating the presence of the NT surface; **b** cross-section view of the NTs; **c** top-view of the NTs by TEM, showing a well-defined diameter; and **d** Ti6Al4V control surface by SEM

**Fig. 2:**
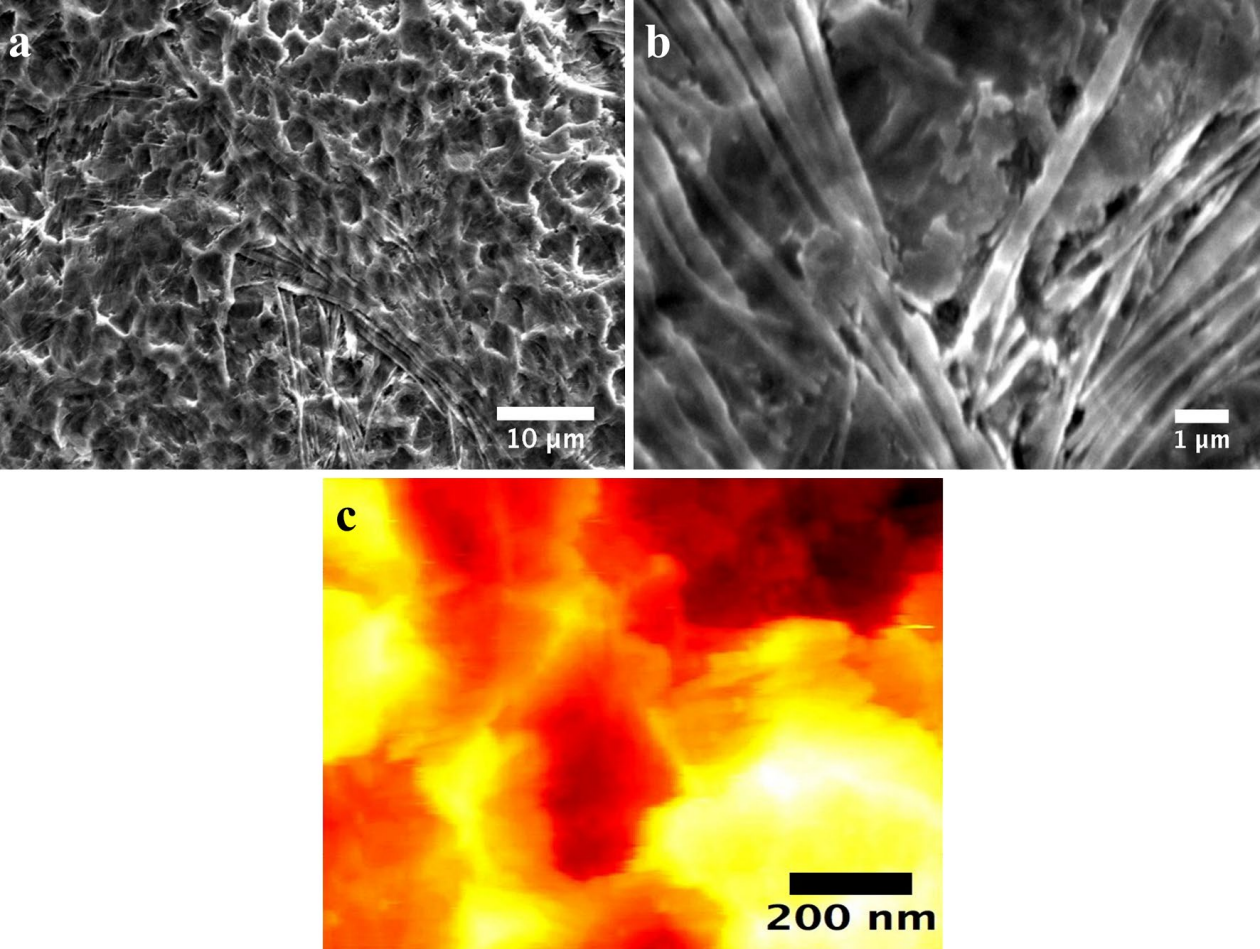
Surface characterization of the acid-etched rough Ti6Al4V surface. **a** FE-SEM micrograph illustrating a low magnification view of the rough material; **b** high magnification of the etched surface showing irregular patterns of valley-peaks with stretch marks; and **c** AFM micrograph representing the rougher and asymmetrical surface

**Fig. 3:**
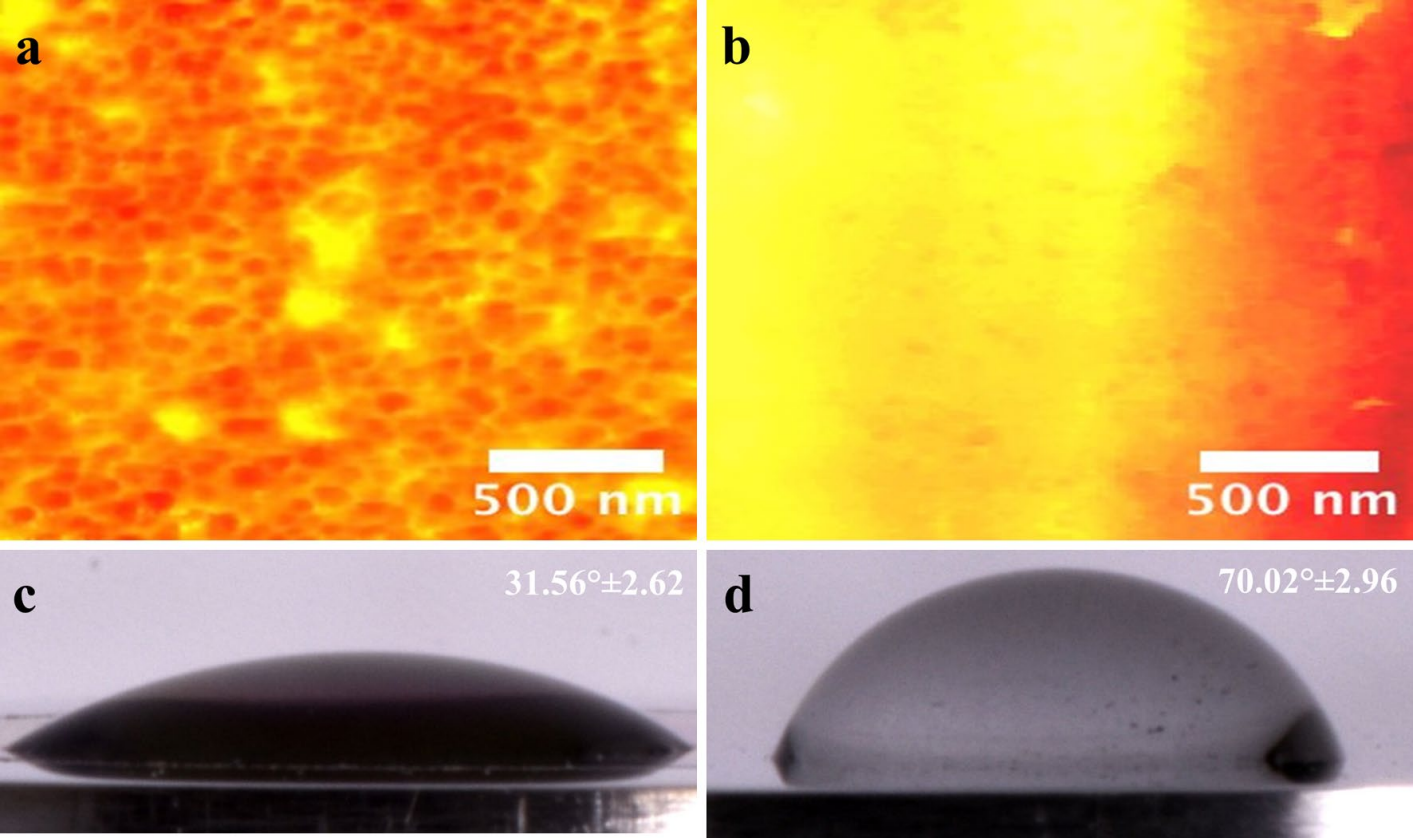
AFM micrographs of the Ti6Al4V surfaces and water contact angle measurements. **a** Ti6Al4V surface with NTs illustrating a rougher surface; **b** Ti6Al4V control showing a smooth and flat homogenous surface, **c** Ti6Al4V with NTs exhibiting a hydrophilic surface; and **d** Ti6Al4V control displaying a hydrophobic surface

Figure 1 presents the surface structural morphology, and SEM analysis reveals the evident formation of ordered Ti6Al4V-NTs on the surface after the anodization process, as expected (Fig. 1a). Moreover, the development of self-organized and well-aligned Ti6Al4V-NTs (Fig. 1b) of approximately 361 nm in length (Table 1) was detected. TEM analysis (Fig. 1c) confirms the abovementioned trends, showing the presence of Ti6Al4V-NTs with an estimated diameter of around 70 nm (see Table 1). On the other hand, the Ti6Al4V alloy presents a flat and smooth surface as predicted (Fig. 1d). Similar outcomes on the surface properties were detected for the cp-Ti based materials. For the acid-etched alloy (see Fig. 2), FE-SEM study showed the formation of a rougher surface with irregular patterns of valley-peaks with extensive presence of reproducible craters Fig. 2a, b. Interestingly, surface roughness is an important parameter that is closely related with the control of the biological properties of biomaterials [36], so the surface roughness and topography of the experimental materials were evaluated, see Figs. 2 and 3. In Fig. 3a it is possible to see an increased surface roughness of 19.19 ± 0.22 nm (see Table 1) on anodized Ti6Al4V-NTs, as depicted by the increased formation of nanostructured dots observed by AFM. These data correlate with the nanotubular morphology presented by the FE-SEM and TEM analysis (Fig. 1a, c). In contrast, the Ti6Al4V alloy surface topography presented in Fig. 3b reveals the presence of a flat and smooth surface (3.71 ± 0.68 nm). Furthermore, the cp-Ti and cp-Ti-NTs materials presented similar topographies as compared to the alloy based materials (data not shown); as they were processed using the similar protocol performed for the alloy material. Additionally, the Ti6Al4V roughed alloy presented an increased surface roughness (190.66 ± 2.66 nm) showing the strict formation of irregular patterns (Fig. 2c) as observed by the FE-SEM analysis. Importantly, it has been reported that surface wettability plays a pivotal role in materials biocompatibility. Thus, in order to characterize this critical property the water contact angle has been evaluated (Fig. 3). As illustrated by the analysis, it is found that the Ti6Al4V-NT surface possessed a lower contact angle 31.56° ± 2.62 (Fig. 3c) in comparison to the flat and smooth Ti6Al4V surface 70.02° ± 2.96 (Fig. 3d). Similar parameter of surface properties (wettability and roughness) detected on Ti6Al4V and Ti6Al4V-NTs were found on the cp-Ti and cp-Ti-NTs.

**Table 1:** Physical surface features of the experimental substrates

Surface	RMS^a^ (nm)	NT diameter (nm)	NT length (nm)
Non-anodized Ti6Al4V	3.71 ± 0.68	N/A	N/A
Ti6Al4V-NTs	19.19 ± 0.22*	70 ± 6.4	361 ± 13.1
Ti6Al4V-rough	190.66 ± 2.66**	N/A	N/A

The differences in RMS between the substrates illustrate an increase in surface roughness after anodization. The NT diameter and length were obtained from TEM and FE-SEM analyses respectively. Values are mean ± SD, n = 3

*, ** p < 0.05 compared to non-anodized Ti6Al4V and Ti6Al4V-NTs respectively

^a^Data obtained from 4 μm^2^ AFM scans

On the other hand, chemical analysis (Table 2) shows an increased oxygen content (25.51%for Ti6Al4V-NTs and 22.34% for cp-Ti-NTs) after anodization on both materials, while EDX was not able to detect the presence of oxygen on the control alloy. Similarly, a slight increase in fluoride (3.18% for Ti6Al4V-NTs and 3.04% for cp-Ti-NTs) and decrease in the carbon content (4.00% for Ti6Al4V-NTs and 3.18% for cp-Ti) was appreciated on the NTs in comparison to the control materials.

**Table 2:** Surface elemental composition by EDX analysis

Surface	Atomic concentration (%)					
	C	V	Al	Ti	O	F
Non-anodized Ti6Al4V	5.96	5.67	5.37	83.00	0.00	0.00
Non-anodized cp-Ti	3.22	0.00	0.00	94.60	2.18	0.00
Ti6Al4V-NTs	4.00	0.00	5.40	61.91	25.51	3.18
Cp-Ti-NTs	3.88	0.00	0.00	73.78	22.34	3.04

### Endothelial characterization

**Fig. 4:**
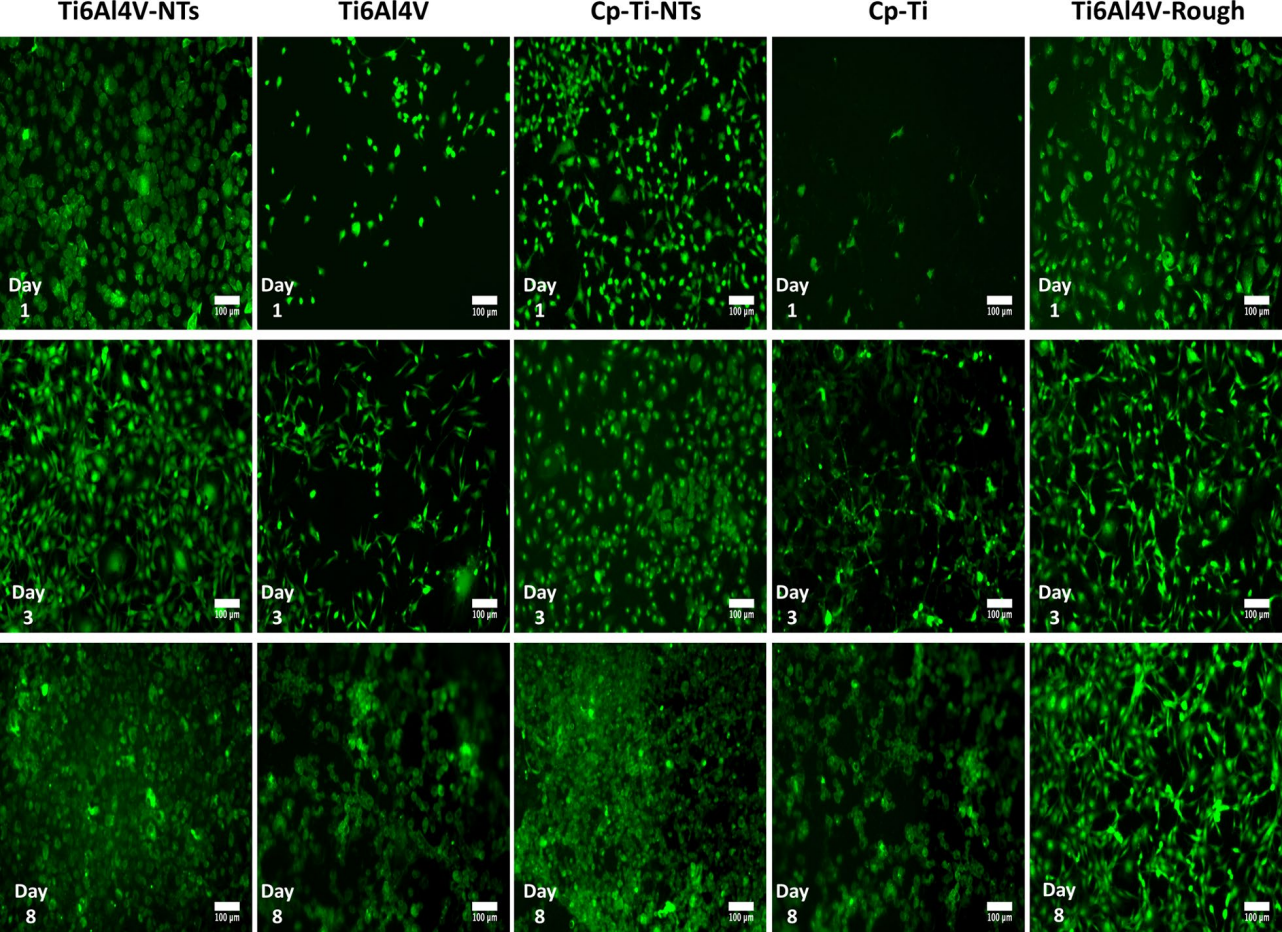
Live/dead stain of BCAECs representing the cell proliferation process and viability status on the material surfaces at different incubation times. It is denoted an increased number of viable cells along the nanostructured surface rather than the non-modified materials

As a primary and determinant process to be induced on dental implant materials, it is important that new recruited endothelial cells have the capacity to vitally grow and proliferate over the surface [7]. Thus, endothelial proliferation and viability have been evaluated on the experimental materials at each culture time, see Fig. 4. For the Ti6Al4V-NTs surface, an evident increase in viable cells was observed at day 1 of culture, when compared to the Ti6Al4V alloy and cp-Ti surfaces. Interestingly, the presence of dead cells (red) was not detected on the different surfaces at this culture point. Moreover, on the cp-Ti-NTs material we did not find outcomes of reduced growth of vital cells after comparing to the nanostructured alloy materials. Furthermore, the rougher alloy presented s similar behavior as to that of the nanostructured materials. Similarly, the cp-Ti-NTs evoked major endothelial growth than the non-nanostructured flat materials. Additionally, at day 3, a sustained propagation of endothelial cells was seen on the NTs and Ti6Al4V-rough surfaces. In contrast, a smaller number of cells were detected on the Ti6Al4V alloy and the cp-Ti. Likewise, at day 8 of cultivation a higher viable cell number was observed growing on the Ti6Al4V-NTs and cp-Ti-NTs surfaces in comparison to Ti6Al4V-NTs and cp-Ti-NTs at day 1 and 3, suggesting cellular proliferation on those surfaces. More importantly, a higher emerging number of viable cells were found on the NTs than on the flat metals. Nonetheless, at this culture point, there was a progressive increase in the cell number on the flat surfaces up to day 8, but this was lower than on the nano-surfaces. Interestingly, the Ti6Al4V-rough surface performed the endothelial propagation with similar results that the nanostructured materials, which may suggests that a rougher surface is in part involved in the growth of endothelial cells.

**Fig. 5:**
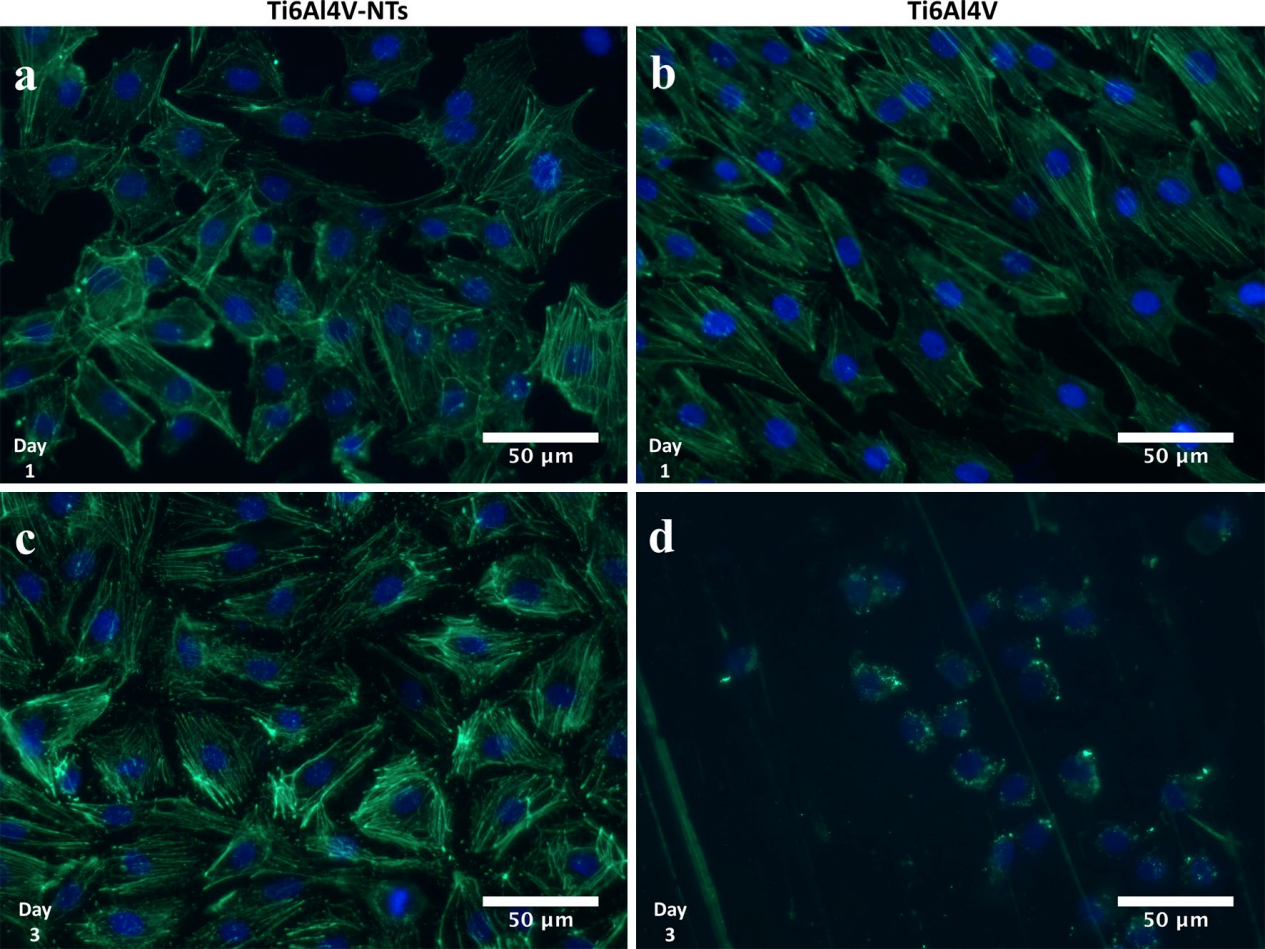
Fluorescence detection of F-actin in BCAECs cultured on the experimental materials for 1 and 3 days. **a** NTs showing a well spread cellular morphology with the evident presence of cytoskeleton stress fiber at day 1 of culture; **b** Ti6Al4V control surface at day 1 illustrating an aligned cellular growth but with a lower cellular spreading; **c** NTs at day 3 displaying the presence of well-defined actin stress fibers; and (**d**) Ti6Al4V control surface showing an altered cellular morphology with the absence of stress fibers

**Fig. 6:**
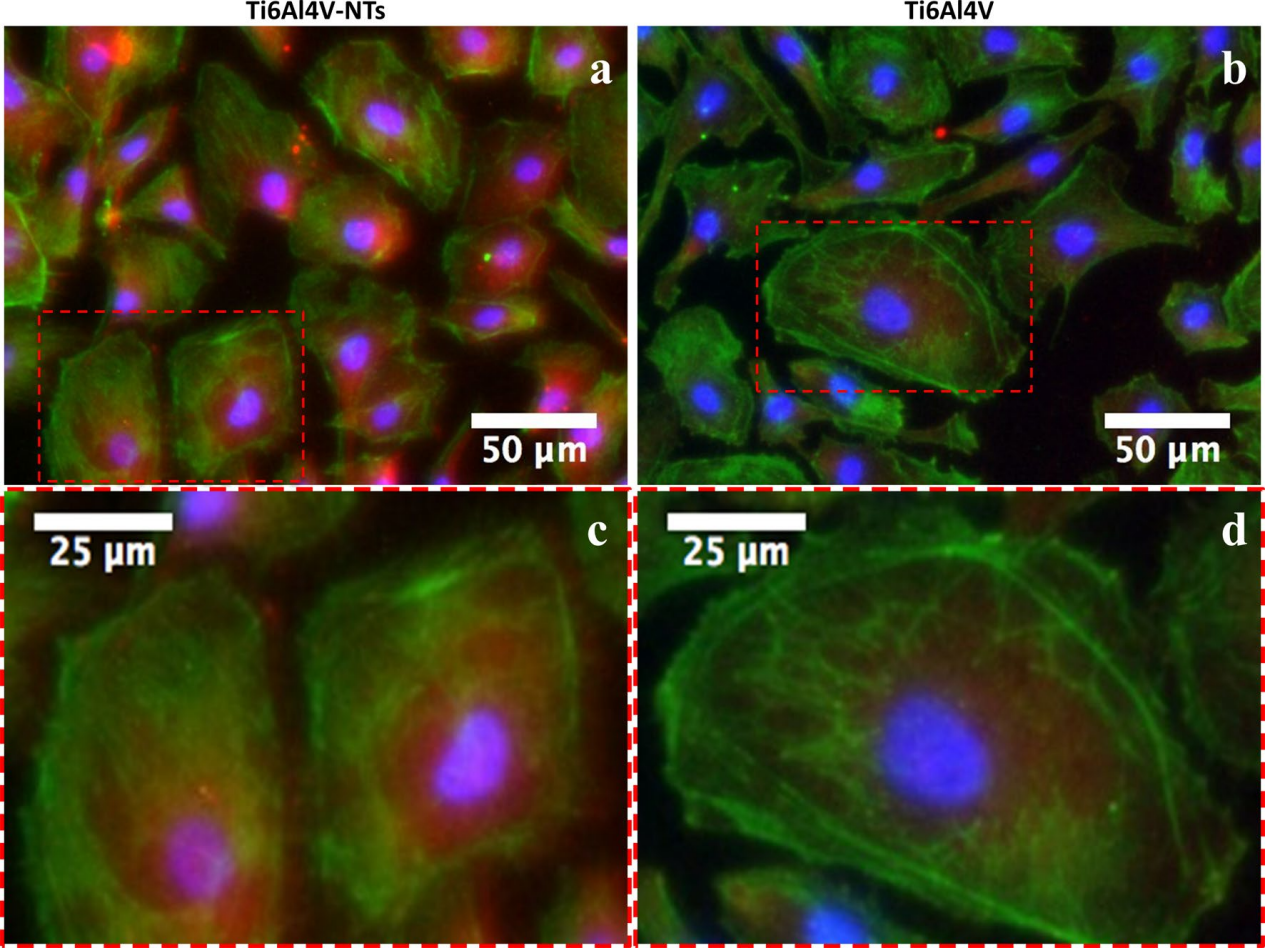
Immunofluorescence analysis representing the staining pattern of vinculin antibody (*red*) with F-actin staining (*green*) among the Ti6Al4V surfaces at 24 h of incubation. **a** BCAECs on Ti6Al4V-NTs illustrating the expression of vinculin among the cells; **b** endothelial cells on flat Ti6Al4V showing a minor expression of vinculin; **c** high magnification of the *red dotted squad* on Ti6Al4V-NTs highlighting the convergence of vinculin and the actin fibers at the membrane zone; and **d** high zoom of the *red dotted squad* on Ti6Al4V presenting the cytoplasmic expression of vinculin

In order to evaluate the cellular morphology in terms of cell spreading and the formation, distribution and structure of F-actin stress fibers on the experimental materials, immunostaining was performed at 1 and 3 days of incubation. At day 1 the presence of adhered endothelial cells was detected on the Ti6Al4V-NT surface, showing a flat and spread morphology along with the formation of cellular interconnections with the existence of well-defined stress fibers, see Fig. 5a. On the other hand, at this exact incubation time on the control Ti6Al4V alloy the presence of well-aligned endothelial cells with the manifestation of actin stress fibers was found (Fig. 5b), but no evident cellular interconnections were appreciated compared to the NT surface (Fig. 5a). At day 3 of cell incubation the irrefutable formation of stress fibers among the NTs was strongly shown (Fig. 5c). Moreover, the cellular morphology on the NTs was more rhomboid-like compared to the NTs and Ti6Al4V alloy in day 1. In contrast, at this exact time the cells cultured on the Ti6Al4V alloy denoted an aberrant cellular morphology (Fig. 5d). Nonetheless, the presence of adhered cells was elucidated, as evidenced by nucleus staining with DAPI on the flat Ti6Al4V alloy surface. Importantly, the presence of vinculin a receptor dependent protein was confirmed and compared among the alloy-based materials. As presented in Fig. 6, at 24 h the Ti6Al4V-NTs stimulated the expression and convergence of vinculin among the endothelial cells (Fig. 6a); meanwhile, the flat alloy also support the presence and action of vinculin although, with reduced expression as suggested by the high magnification image (Fig. 6d). On the other hand, the nanotubular alloy supported the convergence between the F-actin filaments and vinculin as observed at the periphery of the cell (Fig. 6c).

**Fig. 7:**
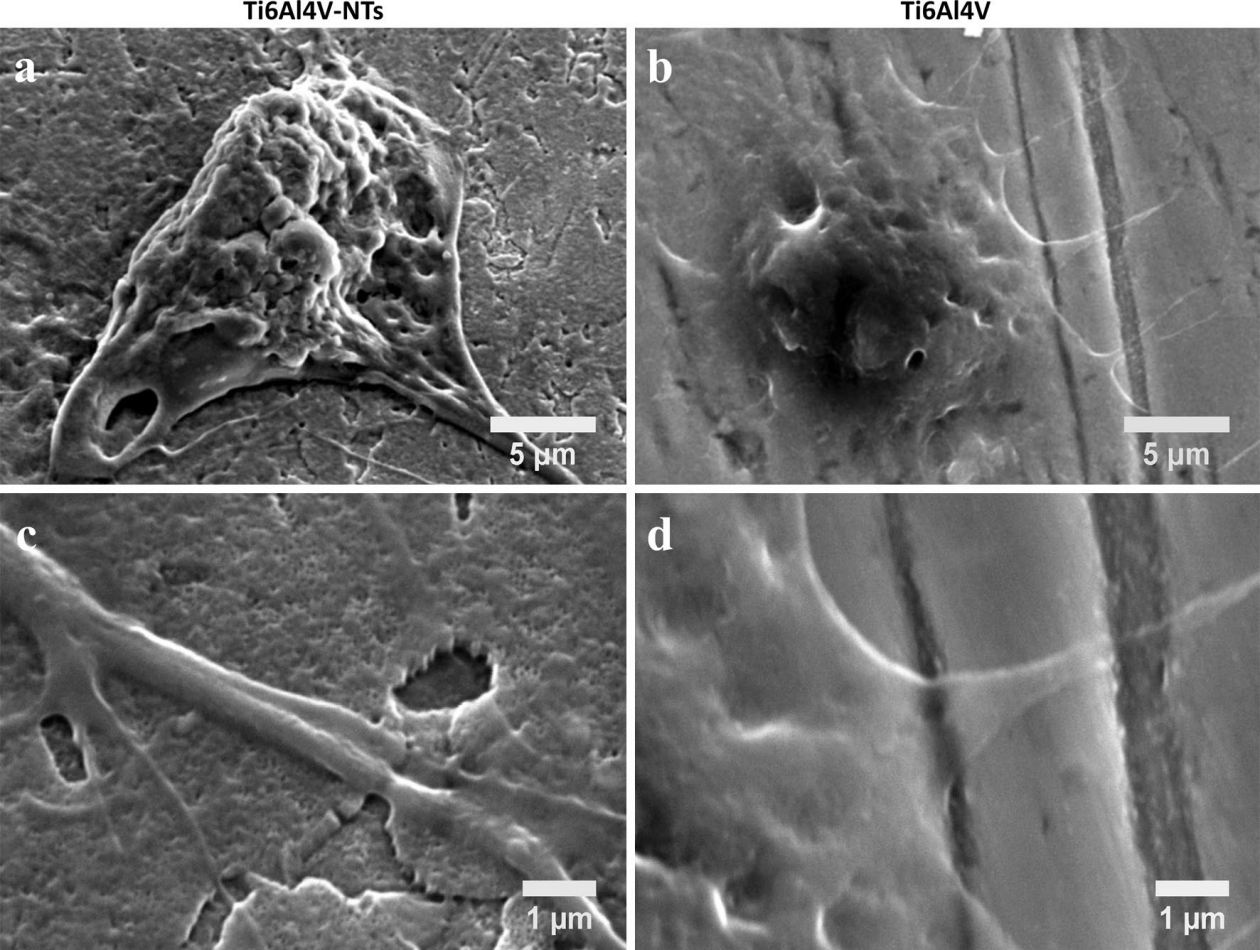
SEM analysis showing BCAEC morphology after 4 h of cell culture. **a** Endothelial cells adhered on the NT surface representing a well-defined cell body; **b** Ti6Al4V control surface illustrating a poorer filopodia formation; **c** higher magnification denoting the configuration of a thicker filopodia anchored to the NTs; and **d** high magnification of Ti6Al4V surface indicating a translucent and impaired filopodia

Cellular adhesion is an essential process required for the formation of new tissue around implants. Therefore, the BCAEC adhesion process was evaluated at 4 h by SEM analysis on alloy-based materials, see Fig. 7. As depicted by Fig. 7a, more evident cellular adhesion and formation of well-anchored and organized cellular bodies was observed on the Ti6Al4V-NTs than on the Ti6Al4V alloy (Fig. 7b). On the counterpart, a translucent cell body was detected over the control surface. Figure 7c evidenced pronounced and significantly elongated protrusions of filopodia with a high degree of contact with the NTs. On the other hand, Fig. 7d illustrates (at the same magnification zoom) translucent and more poorly defined filopodia with a lower grade of contact to the surface, suggesting an inferior adhesion process as observed for the NTs.

**Fig. 8:**
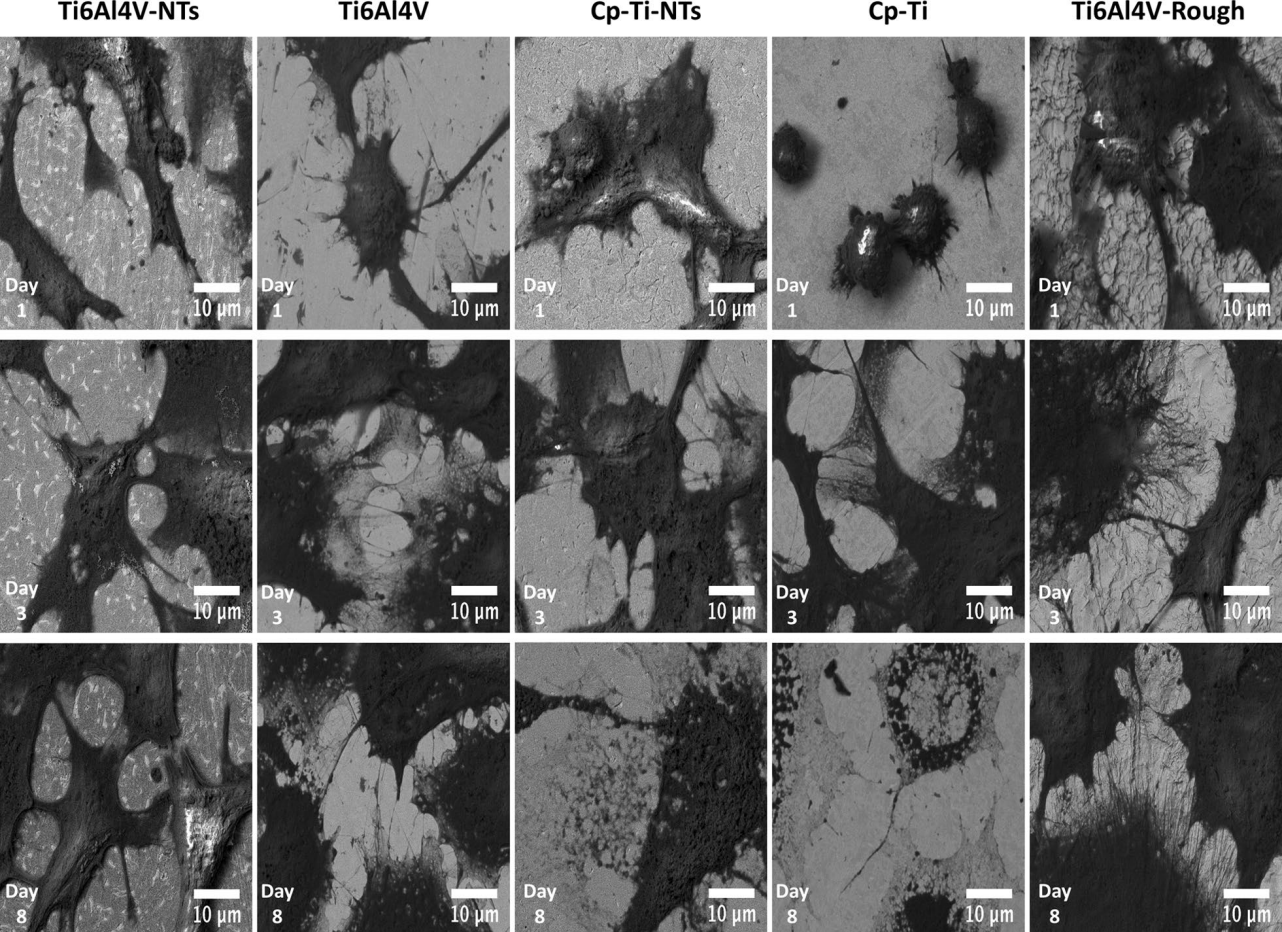
FE-SEM micrographs representing different growing phases on BCAECs at different culture times. It can be highlighted promoted cellular body formation and filopodia extension among the nanostructured Ti6Al4V and cp-Ti surfaces

Endothelial proliferation and morphological organization on the experimental materials is presented in Fig. 8. At day the adhesion of BCAECs on the Ti6Al4V-NTs, showed strikingly higher number and thicker formation of cellular filopodias compared to the Ti6Al4V alloy surface. Moreover, the cp-Ti-NTs and the rougher alloy illustrated outcomes of endothelial propagation with similar formation of thicker filopodia. Moreover, at this culture point it is possible to highlight the evident greater deposition of ECM and cellular interconnections with protrusions on the whole NTs and rough alloy, while on the non-modified surfaces we were not able to identify any important change, showing a circle-like morphology with poorer bonding filopodia. This information suggests a protagonist role for the NTs and surface roughness in the control of the early endothelial proliferation process. Additionally, after 3 days of BCAEC incubation on NTs materials a sustained formation of cellular interconnections takes place, with cells spreading along the surface and the presence of cell bodies with cellular edges, suggesting a monolayer-like formation. Similarly we detected the generation of a high number fipolodia on the rough alloy; however, cells displayed shorter extensions than on the NTs surfaces. An evident cellular proliferation is also recognized on the NTs in comparison to day 1 in both experimental materials. Besides, the cells grown on the Ti6Al4V alloy displayed poorer cellular proliferation, with a flat and poor-spread morphology and lacking cellular bodies on the material surface. Similar results were characterized for the smooth cp-Ti surface. Furthermore, at this incubation interval the NT surface advocates itself as a preferable configuration for optimal endothelial propagation on the Ti-based surfaces. After an extended incubation period of 8 days, significant cellular proliferation is detected on all the NTs, elucidating the remarkable presence of cell bodies, cellular edges and complete interconnections between the entire cells and the surface. In contrast, the cells incubated on the flat materials showed similar behaviors like the status at day 3 with decreased proliferation, a lower number of cell bodies, poorer cellular edges, lower surface coverage, and a continuing aberrant morphology compared to the NTs. The Ti6Al4V rough alloy continued generating a great number of cellular anchorages.

**Fig. 9:**
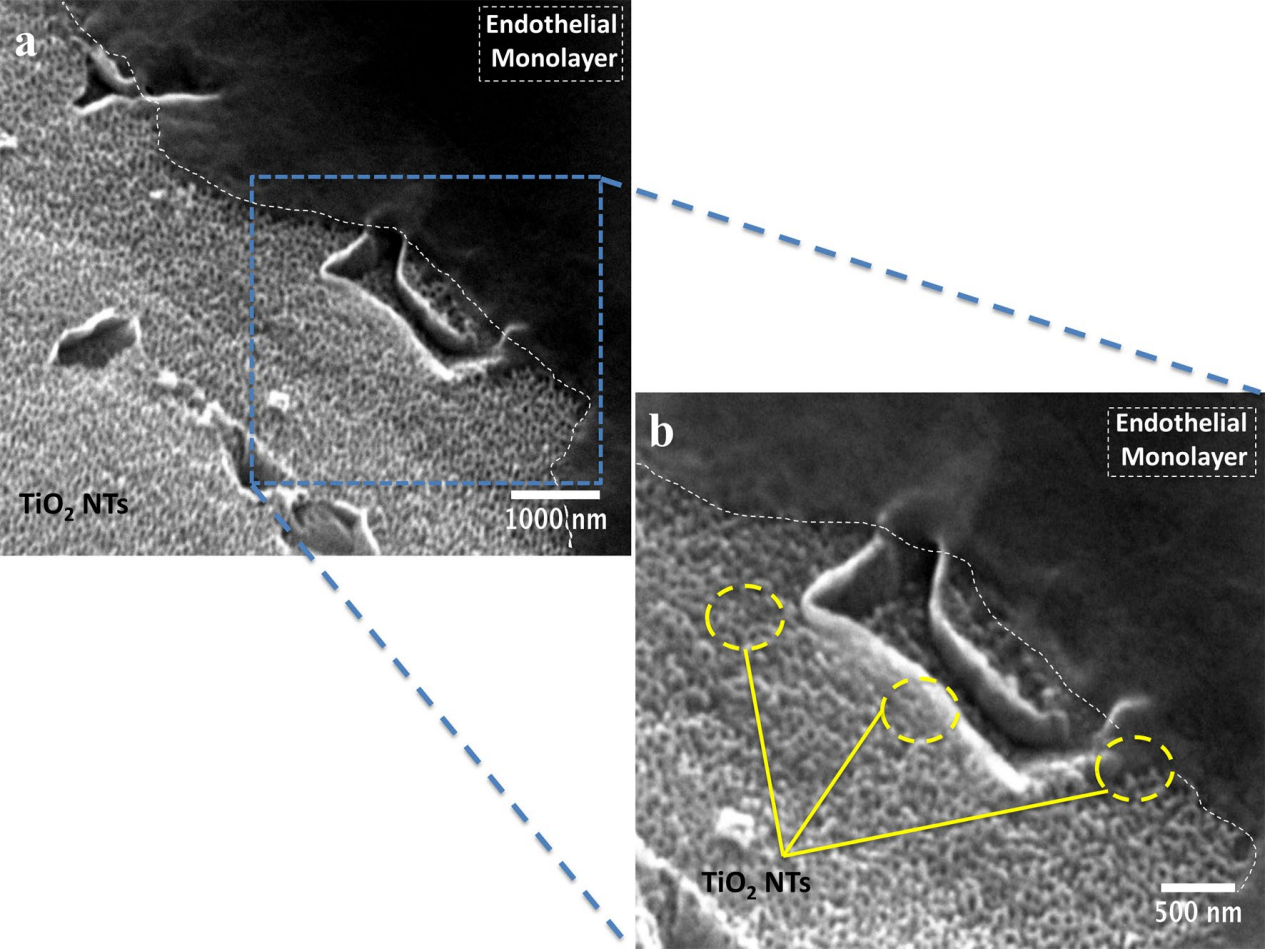
SEM micrographs highlighting the formation of endothelial monolayer on the NTs in a cross-section view after 24 h. **a** Endothelial layer (*white dotted lines*) growing on the NT surface at low magnification; and **b** Endothelial monolayer (*white dotted lines*) spread on the NTs (*yellow circles*) at higher magnification

Figure 9 highlights the evident formation of the important endothelial monolayer around the Ti6Al4V-NT surface, suggesting a well functionalized cellular morphology guided by the nanostructured material (see Fig. 9a). Moreover, it is relevant to mention that the endothelium exists as a flat and stretched monolayer that is conducted by the growing surface [37], so attention was paid to the monolayer organization and its intimal contact with the NT surface, showing evident monolayer-like formation and suggesting the favorable microenvironment of the NTs as depicted by Fig. 9b.

**Fig. 10:**
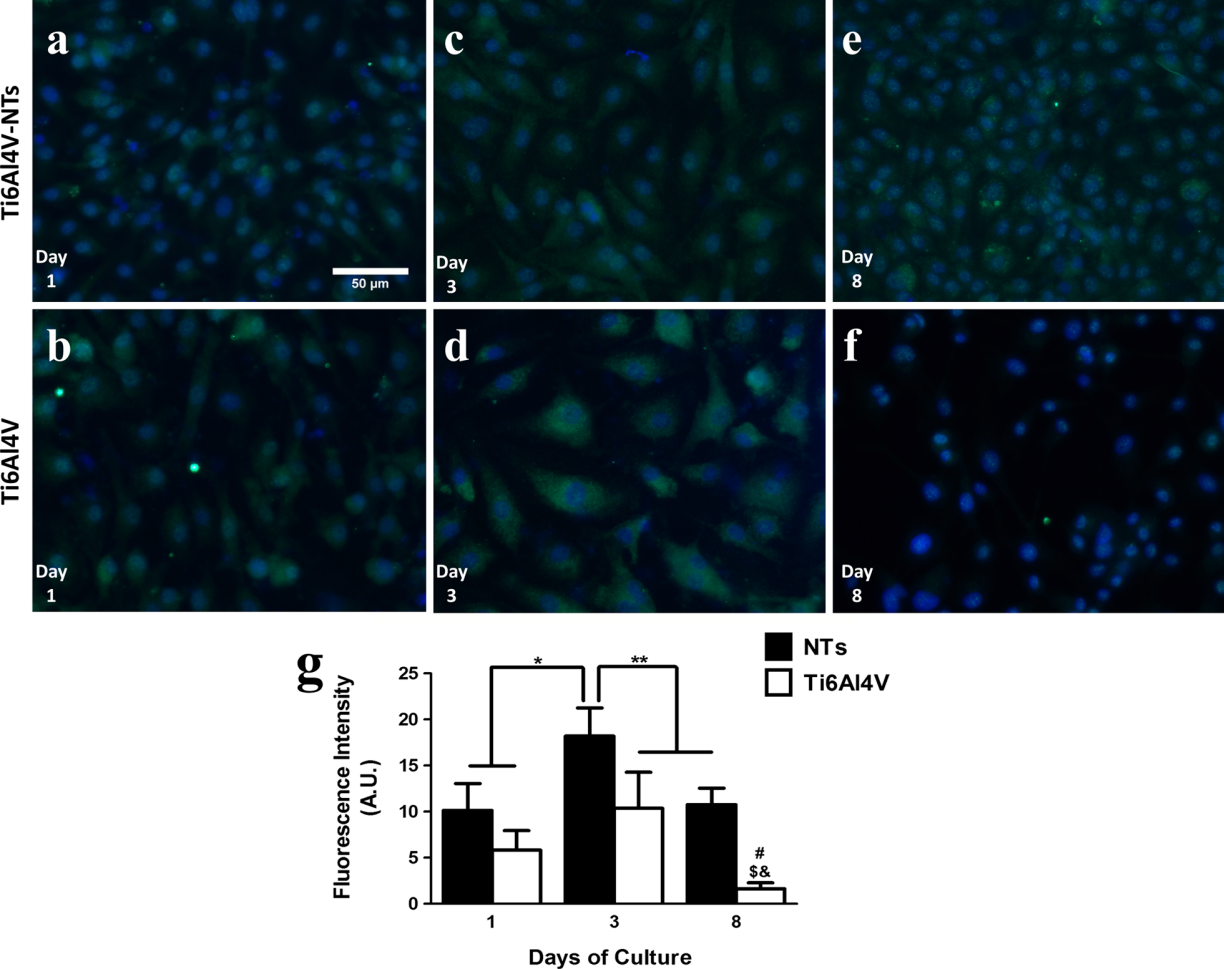
Immunofluorescence of p-eNOS expression in BCAECs cultured on the experimental surfaces. **a** BCAECs on the NT surface at day 1 of culture; **b** Ti6Al4V surface with endothelial cells at day 1; **c** NTs with BCAECs at day 3 of cell growth; **d** BCAECs on flat Ti6Al4V surface at day 3 of incubation; **e** NT surface with BCAECs at day 8 of cell seeding; **f** Ti6Al4V control surface at day 8 of cultivation; and **g** graph representing the cellular fluorescence intensity expressed for p-eNOS as a function of time. Values are mean ± SD, N = 5. *Denotes differences between NT surface at day 3 versus day 1 of culture on NTs and Ti6Al4V alloy. **Illustrates significant changes for NTs at day 3 compared to Ti6Al4V at day 3 and for the NTs and Ti6Al4V group at day 8 of culture. ^#^ Showed remarkable differences for NTs and Ti6Al4V at day 8. ^$^ Represents striking divergences between Ti6Al4V at day 8 and Ti6Al4V at day 3 of growth and demonstrates important contrasts after comparing Ti6Al4V at day 8 versus day 1

As protagonists of endothelial function and angiogenesis, the presence of the phosphorylated/activated form of a key protein involved in these processes, p-eNOS, was evaluated on the alloy surfaces. Figure 10 illustrates the expression of p-eNOS in BCAECs. It is possible to observe the intracellular and cytoplasmic localization of p-eNOS, mainly confined around the nucleus for each surface at all the incubation times (refer to Fig. 10a–e). However, at day 8 there was a lack of any visible p-eNOS staining on the cells cultured on the Ti6Al4V alloy (Fig. 8f). Thus, in order to accurately evaluate and compare any significant differences between the surfaces at each incubation time, a representative graph is given for the p-eNOS expression (Fig. 10g). At day 1 of culture, an increased expression we evaluated on the NT surface, but with no significant differences between the two materials (*P* > 0.05). Moreover, the p-eNOS expression increased considerably after 3 and 8 days of culture on the Ti6Al4V-NT surface when correlated with the Ti6Al4V alloy and for NTs and flat Ti6Al4V at day 1 and 8 respectively (*P* < 0.05). Interestingly, p-eNOS staining was reduced at day 8 compared to day 3 in cells cultured on NTs, but showed a similar expression compared to the cells on NTs at day 1. Notably, a striking reduction in p-eNOS was evidenced at day 8 in cells growing on the Ti6Al4V alloy control.

**Fig. 11:**
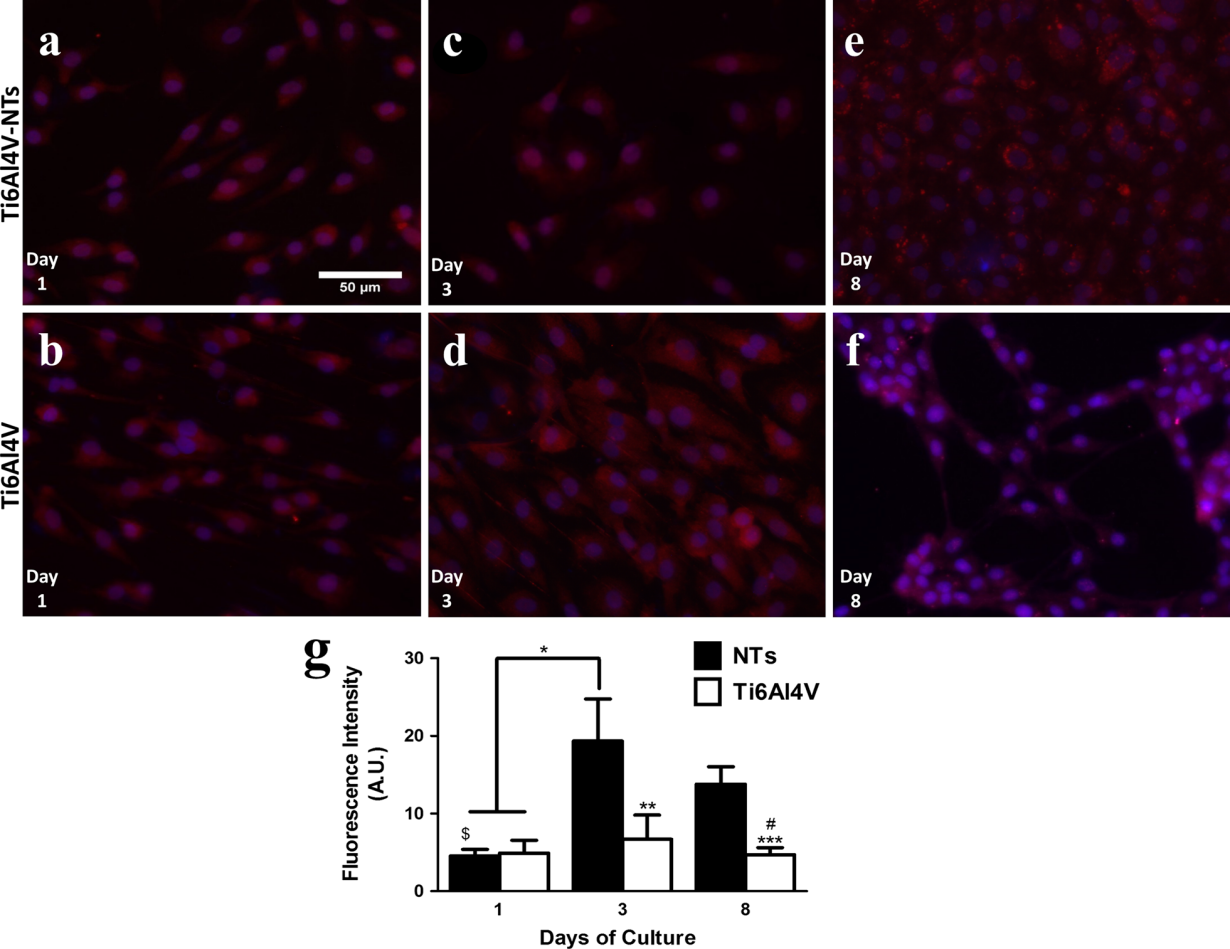
Immunolabeling of p-VEGFR2 expression in BCAECs cultured on the experimental surfaces. **a** BCAECs on the NT surface at day 1 of culture; **b** Ti6Al4V surface with endothelial cells at day 1; **c** NTs with BCAECs at day 3 of cell growth; **d** BCAECs on Ti6Al4V flat surface at day 3 of incubation; **e** NT surface with BCAECs at day 8 of cell seeding; **f** Ti6Al4V control surface at day 8 of cultivation; and **g** Graph of the cellular fluorescence intensity used to measure the expression of p-VEGFR2. Values are mean ± SD, N = 5. *Denotes differences between NT surface at day 3 versus day 1 of culture on NTs and Ti6Al4V alloy. **Illustrates significant changes for NTs at day 3 compared to Ti6Al4V at day 3 and for the NTs. ***Represents statistical differences between NTs and Ti6Al4V at day 8 of incubation. ^$^Denotes important divergences after analyzing NTs at day 1 and NTs at day 3 and 8. ^#^ Remarkable contrast between Ti6Al4V at day 8 and NTs at day 3

VEGFR2 is one of the primary receptors responsible for binding VEGF to endothelial cells, thereby resulting in the promotion of angiogenesis [38], as required for the vascularization success of dental implants. Thus the activation of VEGFR2 was studied on the alloy surfaces and is presented in Fig. 11. As represented by the fluorescence micrographs, it can be highlighted that the phosphorylated form of VEGFR2 was mainly distributed among the cytoplasmic region of the endothelial cells on both experimental materials (see Fig. 11a–f). This suggests a similar pattern to those observed in p-eNOS. For accurate assessment of p-VEGFR2, the staining was quantified and is provided as a representative graph (Fig. 11g). After 3 and 8 days of cell incubation on NTs a significant expression of p-VEGFR2 was observed compared to the Ti6Al4V alloy at day 3 and 8, and on both materials cultured for 1 day Additionally, a decrease was seen in p-VEGFR2 staining on the NTs at day 8 compared to day 3, but these differences were not significant.

## Discussion

Ti-based alloy materials especially Ti6Al4V are the main option selected for the manufacturing of dental implant screws. In an endeavor to stimulate the osteogenesis process on these surfaces, different modification techniques such as acid-etching, plasma-spraying and even grit-blasting have been applied [10–12, 39]. However, these techniques present a number of clinical and physicochemical drawbacks that are detrimental to their mechanical, biological and clinical performance [11]. On the other hand, angiogenesis, the process that involves the generation of new blood vessels, is a pivotal requisite for the promotion and sustainability of the osseointegration required on dental implants [40]. In fact, several studies have demonstrated that insufficient angiogenesis or inadequate bone vascularization after early bone implantation will result in insufficient oxygen supply, inducing hypoxia and cellular necrosis [41–43]. This negative consequence will lead to impaired bone regeneration by the formation of fibrous tissue, ultimately causing the loss of the implant. Furthermore, Ti6Al4V dental implants may support the angiogenesis process due to the osseointegration process observed in several clinical treatments, mechanism which in turn requires an angiogenic activity. Moreover, angiogenesis is necessary for adequate tissue remodeling, bone healing and wound healing over metallic scaffolds. Thus, in order to attain this elusive goal, emerging nanostructured materials have been studied as promising options to address this important problem. It is notable that most of the existing work regarding nanostructured biomaterials has focused mainly on the role and function of bone-forming osteoblasts, rather than the role of endothelial cells.

NTs have evidenced promising properties as stimulating surfaces for the adhesion, proliferation and maturation of different types of osteoblasts [44–46] when compared to flat or even microstructured surfaces as observed here. The authors have shown previously that osteoblasts and even chondrocytes cultured on our NTs are highly improved. Thus, in the present study these NTs are further evaluated in order to achieve proper in vitro endothelialization, as a requirement for well-induced osseointegration. As previously reported [14], our simple and rapid anodization method successfully synthesizes NTs with a diameter of ~70 nm and a thicker TiO_2_ coating (~360 nm). This paper now provides additional data (not previously reported in the NT synthesis method) including the appliance of this anodization technique on cp-Ti and also the water contact angle (31.56° ± 2.62) of Ti6Al4V-NTs. A contact angle smaller than 65 is commonly considered a hydrophilic surface, and higher than 65 is considered to be hydrophobic [47]. This information suggests that our NTs have improved surface hydrophilicity. It is well-known that nanostructured surfaces may present increased surface roughness and even high hydrophilicity [19, 20]. For instance, Roguska et al. [19] synthesized NTs applying different voltages and consequently reported decreased water contact angle measurements for their different NTs in comparison to a Ti control surface. Zhang et al. [20] suggested a significantly lower water contact angle for NTs with ~360 nm thickness when evaluated versus a non-modified Ti surface. These authors also described increased surface roughness for their NTs, information that strongly agrees with our NTs. Interestingly, in our research a higher percentage of oxygen is detected after the EDX analysis on both NTs compared to the control surface. This striking change may be due to the increased coating thickness that was generated after the anodization process, supporting the results observed by SEM analysis. Moreover, a reduction in the carbon (C) percentage was observed after performing the anodization and cleaning protocol. This observation may in part be supported by the significantly decreased water contact angle seen for the NTs surfaces, and may also be explained by the cleaning process and the UV used for sterilization, as suggested by others [18, 45, 48]. It is worth noting that anodization is usually carried out using constant voltages in water solutions containing fluoride as electrolyte. This suggests an initial dissolution of the surface by the transportation and migration of fluoride ions, giving rise to Ti reduction into Ti^4+^ which forms a stable complex such as [TiF_6_]^2−^ species [49]. This model suggests being in line with our results due to the increment in the F content found on NTs compared to the non-anodized alloy surface.

A key process involved in the formation of new blood vessels and capillaries is the capability of viable endothelial cells to grow and proliferate in response to different factors, such as the surface of an implant [7, 50]. For this reason, the proliferation of endothelial cells was first evaluated by a live/dead stain, showing enhanced cellular growth on the NTs surfaces and on the roughed Ti6Al4V alloy for all the incubation times compared to the non-treated materials. This interesting trend may be explained in part by the decreased water contact angle seen on the NTs compared to the smooth surfaces. As a lower water contact angle means higher hydrophilicity, this parameter could be translated into promoted interactions involving electrostatic forces, hydrogen bonding and van der Waals forces [51]; leading to promoted binding of water molecules and salt ions [52]; thereby resulting in a developed affinity of the surface to the initial coating of different proteins (e.g. fibronectin or vinculin) controlling the self-assembly, conformation and atomic structure of ECM proteins that must be synthesized and deposited over a surface for proper cellular proliferation [53–55]. Moreover, in a agreement with our results, Kopf et al. [51] reported that the chemical wettability of SLActive surfaces (a rougher Ti surface such as rough Ti6Al4V, presented herein) combined with nanostructures, significantly improved the adsorption of proteins when compared to hydrophobic rough Ti, suggesting that the presence of a nanosurface could potentiated the coating of proteins. In the same way, the nanoconfigured surface also showed a significantly rougher surface compared to the flat alloy, a property that has been widely evidenced to improve the capability of a material to promote cellular proliferation [56]. In a previous report that compared the endothelial proliferation rate on flat, a sub-micron and a nanomodified Ti surfaces, it was shown that the nanotextured surface presented the highest endothelial proliferation rate at days 1 and 5 [57]. Furthermore, Loya et al. [56] reported an increased endothelial proliferation after 7 days of culture on a rougher and more hydrophilic nanostructured surface than on a flat and non-nanostructured material, whereas here we evaluated the endothelial proliferation up to 8 days of cultured BCAECs with similar results, as described above. Our data further advocates the notion that nanomodified surfaces promote and support initial and late endothelial proliferation. Similarly, the rougher surface sustains the endothelial propagation at all incubation times, with a correlative behavior as to that observed for the NTs; information that proposes an important role of surface roughness in BCAECs growth. Furthermore, it has been hypothesized in the literature that Ti-based alloy materials containing Vanadium could induce a cytotoxic environment by promoting oxidative stress [58, 59]. However, cell viability assays on our Ti6Al4V alloy compared to pure Ti (lacking V) surfaces suggest similar vitality with a lack of toxicity on Ti6Al4V alloy containing different metals (mainly Al and V). Interestingly, an identical trend was detected between the Ti6Al4V-NTs and cp-Ti-NTs. Importantly, the EDS analysis indicated no presence of V on the Ti6Al4V-NTs; which advocate that the main elements responsible for angiogenesis over the nanostructured surfaces could be Ti and O; nonetheless, more chemical and analytical studies are recommended to elucidate this hypothesis. Moreover, taking into context the NTs diameter, previous studies have suggested that NTs among 60–100 nm may favorably advocate a superior endothelial activity and promoted mesenchymal stem cell differentiation into osteoblasts cells [15, 60], as observed in our present study. However, Park et al. [61] evoked a superior endothelial activity among smaller diameter NTs (15–20 nm) instead of larger NTs (60–100 nm). These controversial differences could be due to varied parameters (mouse endothelial cells, VEGF in growth medium, deionized water for anodization and heated-treated and crystallized, anastase phase TiO_2_ NTs in Park et al.’s study versus bovine endothelial cells, non-VEGF in culture medium, SOW for NTs synthesis and as-anodized, amorphous-phase TiO_2_ NTs in ours) among the studies. In order to clarify these findings, more studies regarding the angiogenic activity, different NTs dimensions, culture conditions, anodization method and crystal structure are needed. Furthermore, it is important to highlight that both experimental surfaces (NTs and flat Ti6Al4V alloy) have sustained the presence of viable cells at each incubation time point. However, the number of viable cells cultured on the NT surface was higher than on the non-modified material and similar to the rougher alloy. These findings may be explained in part by the cytoskeletal configuration of endothelial cells that is dependent on the material surface characteristics. As an increased arrangement of criss-cross pattern cytoplasmic stress fibers within cell bodies is involved in cellular locomotion, proliferation and intimately associated to organelle movement and biochemical activity [62], it was detected that this behavior is closely consistent with the endothelial morphology observed on our NTs, whereas the alloy surface only maintained this condition for 24 h (see Fig. 4). Likewise, the presence of active vinculin (a receptor dependent protein) converging with filaments of F-actin, could contributed in the formation of focal contacts and activation of the focal adhesion kinase signaling pathway [63]; a metabolic route that is involved in strong cell adherence to material surfaces and promoted cellular survival and propagation [64, 65] as proposed by our results. These trends may be explained by the fact that NTs are interconnecting networks with a high surface area and porosity that has been suggested to promote and sustain a suitable microenvironment due to the easy transport of nutrients, growth factors and biochemical signals for different kind of cells [62, 66, 67]. Besides, a flat and smooth surface does not offer an adequate cellular environment for proper cellular growth, as has been seen in a number of studies [15, 57, 68].

Cellular morphology during the adhesion process is an important parameter that displays the initial cellular behavior promoted by the interplay between cells and surfaces. A promoted endothelial adhesion was observed among the NTs, as denoted by the striking formation of several cellular filopodias that were lower in the flat Ti6Al4V alloy. As observed in Fig. 7, intimate contact between NTs and cellular filopodia can be suggested, promoting an overall increase in cell-substrate interactions. It is worth noting that NTs are ordered and aligned nanoholes that promote cellular anchorage and migration by filopodia probing. Because this topographical configuration acts to facilitate cellular movement, this may induce a beneficial scenario for important processes such as neovascularization during the initial bone formation and wound healing [15, 43]. Similarly, it has been suggested that faster re-endothelialization is preferred in order to achieve successful endothelial growth and functionality [69].

It is a major challenge during neo-vascularization around dental implants to increase the affinity of endothelial cells for the surface in order to stimulate them to form a protective monolayer after initial implantation and early bone formation. Thus, it is suggested that for proper and healthy endothelial growth it is crucial to form a sustained endothelial monolayer on the surfaces, allowing an intimate endothelial cell arrangement more similar to the natural endothelium [70]. This behavior must furthermore be sustained for a prolonged time period. As observed in Fig. 8, our NTs surface triggered a rapid endothelialization process compared to the flat and smooth materials, completely coating the entire NTs after 3 and 8 days of cell culture and preserving the monolayer morphology on NTs, as evidenced by different analytical techniques.

Activations of angiogenic factors play key roles in the formation of new blood vessel and capillaries. An important factor required for the modulation, proliferation and formation of a healthy endothelial monolayer is the synthesis of NO by the activation of eNOS [71]. As NO is also involved in the control of platelet activity, fibrin deposition and in part in recruitment of inflammatory cells among the endothelial layer, this should be generated for the control of correct wound healing and sustained bone formation after implantation. Indeed, our study shows that the phosphorylation of eNOS was significantly increased on cells cultured on the NTs, suggesting increased enzyme activation and a potentially enhanced endothelial function. This effect may be observed due to the activation of cellular receptors such as mitogen-activated protein kinases (MAPKs), which have been associated with increased activity by nanostructured surfaces [72]. Moreover, these important proteins have been involved in mechanotransduction and sensing of shear stress and associated with the activation of the cell adhesion family proteins α_1_β_1_-integrins and α_5_β_3_-integrins (transmembrane receptors involved in angiogenesis) in endothelial cells [73–77]. Similarly, α_1_β_1_-integrins have been involved in the activation of Src kinase [74], in the Akt/PI3K/eNOS and ERK 1/2 pathways [72, 78]. Moreover, a plausible mechanism could be the activation of α_5_β_3_-integrins by the NTs geometry; as the size of the integrin domain is about 10–20 nm [79], thereby NTs may allow a successful clustering of integrins which could promote the convergence between vinculin and F-actin filaments (as observed in our study). Likewise, TiO_2_ is a chemical stable compound that may not compromise the chemical integrity of integrins, resulting in optimal integrin activation; which is strongly associated to the formation of focal adhesion points, endothelial adhesion, proliferation, stress fiber formation (as demonstrated here) and downstream of the Akt/PI3K/eNOS signaling pathway in endothelial cells [76]. Interestingly, Chaudhuri et al. [76] proposed that single walled carbon nanotubes in contact with endothelial cells are able to activate in vitro and in vivo angiogenesis via Akt/PI3K/eNOS by the phosphorylation of α_5_β_3_-integrins, which in turns induced the activity of focal adhesions (involving Vinculin). It has furthermore been shown that dynamic structural cytoskeletal actin morphologies and the presence of actin stress fibers among endothelial cells may have a positive impact on the activation of eNOS via caveolar membrane domains and caveolar-membrane-associated proteins [71, 80]. On the other hand, it has been acclaimed by Mukherjee et al. [81] that low dosages of graphene oxide and reduced graphene oxide in endothelial cells may promote in vitro angiogenesis via activation of Akt which further increased p-eNOS levels. Thus, based on the aforementioned evidence, this information suggests that angiogenesis on Ti6Al4V-NTs is in part modulated by the above-mentioned pathways, eNOS activity and the cytoskeletal arrangement of F-actin filaments with vinculin expression which promote cell–cell and cell-material adhesion. However, more analytical and molecular analyses are recommended in order to clarify these hypotheses. Another crucial protein that takes part in the angiogenesis process is the activation of VEGFR2 in endothelial cells by the effect of VEGF or by the surface of an implant [7]. As demonstrated here, our NT surface triggered the activation and sustained phosphorylation of VEGFR2 for all the incubation time points, showing a higher rate at day 3 of culture compared to day 8. This effect may be explained in part by a faster initial proliferation rate on the endothelial cells with a subsequent decreased in proliferation [82]. Endothelial cells need a large enough surface area in order to rapidly proliferate over a surface. However, when they reach confluence they enter a stationary phase, thereby down-regulating their proliferation and as a consequence diminishing the expression of proteins associated to endothelial propagation [82, 83]. On the other hand, the activation of VEGFRs, which leads to the synthesis and secretion of VEGF by endothelial and osteoblasts cells, has been suggested to be of pivotal importance in order to promote the angiogenesis and osseointegration process over a material surface [84, 85]. Furthermore, we hypothesize that the promoted adhesion and proliferation of endothelial cells (outcomes of angiogenesis) over our NTs may decrease the intracellular synthesis of reactive oxygen species (ROS) by the induction of a favorable microenvironment due to the NTs, suggesting improved angiogenesis via a VEGF-dependent pathway [86]. Moreover, similarly to the suggested mechanism exerted by e-NOS on angiogenesis; VEGFR2 activation (phosphorylation) has been correlated to the behavior of focal adhesions and the phosphorylation of PI3K [87, 88], proposing that NTs may stimulate the effects of α_5_β_3_-integrins, which in turns promotes focal adhesion complexes with increased stress fiber formation and vinculin expression generating angiogenesis (via VEGF-dependent pathway). For instance, Pezzatini et al. [85] reported an increased expression level of VEGFR2 and eNOS by endothelial cells cultured in contact with nanomodified hydroxyapatite crystals after 10 days of culture, leading to a high production of NO by endothelial cells. Moreover, Gittens et al. [84] suggested increased production of VEGF from primary human osteoblasts cells (HOBs) and mesenchymal stem cells cultured over a hydrophilic nanomodified Ti6Al4V alloy surface, when compared to a smooth and flat surface. This information suggests that cellular contact with nanomodified surfaces may promote an adequate microenvironment to sustain functional endothelial growth, which will result in promoted osseointegration around the implant leading to a successful clinical result.

## Conclusions

**Fig. 12:**
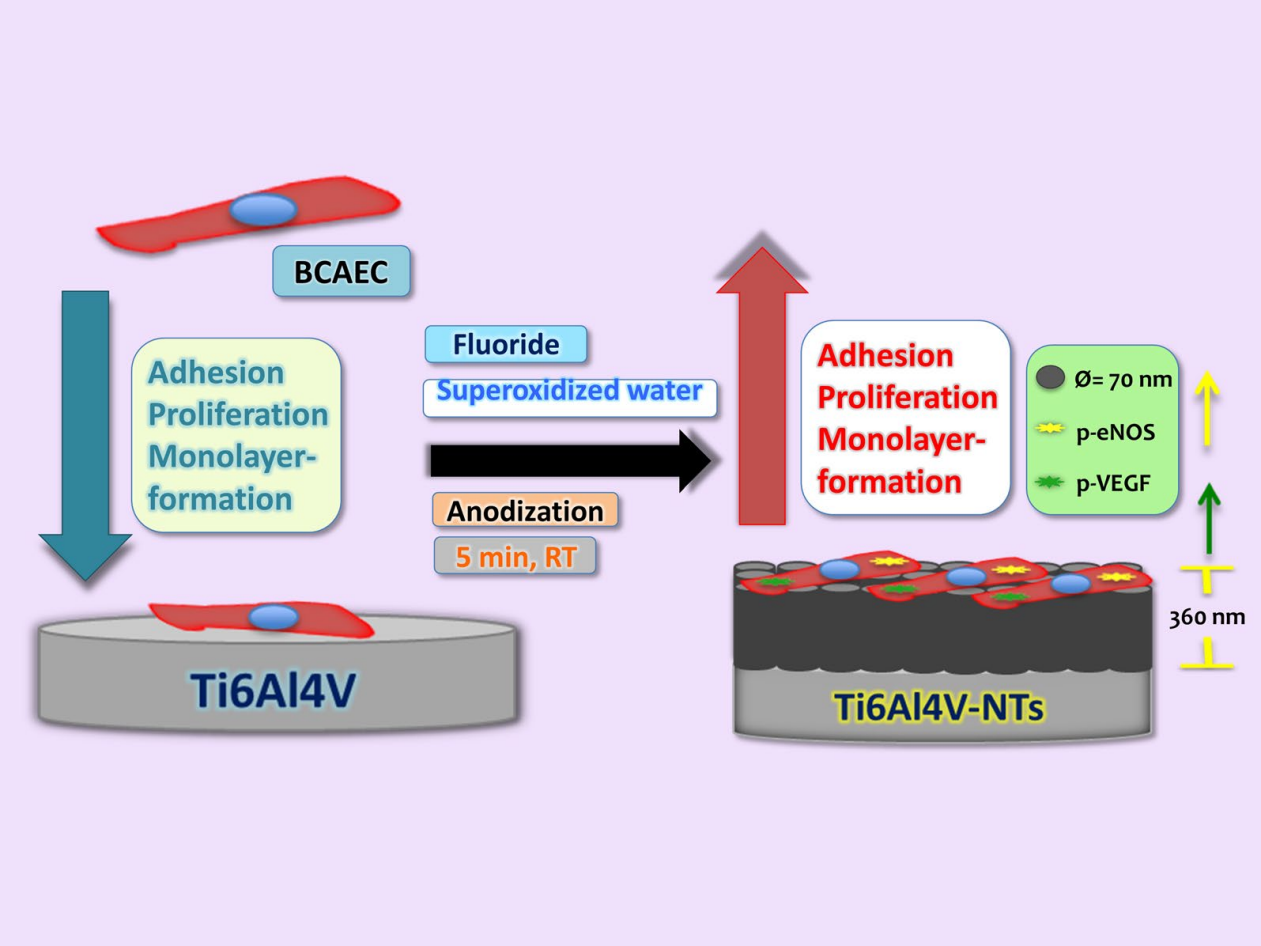
Schematic representation illustrating the effects of 70 nm NTs anodized and cleaned with SOW improving the angiogenic behavior in terms of monolayer formation, viability, proliferation and activation of important endothelial factors compared with a non-anodized Ti6Al4V alloy

The present study shows that Ti-based materials with surface-modified NTs of ~70 nm diameter synthesized with our simple and faster method increases the surface roughness and hydrophilicity of the material, resulting in the promotion of in vitro endothelial behavior with increased activation of angiogenic factors, as schematized in Fig. 12. Based on the results of angiogenesis analysis on cp-Ti and Ti6Al4V alloy, our data suggest, that mainly Ti and O are intrinsically involved in the process of angiogenesis of Ti-based materials. More importantly, our results proposes that rougher surfaces are preferred rather than flat materials, due to the potentiated endothelial propagation that was observed over the acid-etched alloy. This information may open up a path for further research on the use of rough NTs to improve the clinical performance of implant materials. However, more molecular and physicochemical studies are recommended in order to clarify the abovementioned theories postulated here. Similarly, more attention must be paid to the neo-vascularization process around implanted materials and in vivo assays are suggested.

## Abbreviations

NTs: TiO_2_ nanotubes
SOW: super oxidized water
Ti: titanium
cp-Ti: commercially pure Ti
Ti6Al4V-NTs: Ti6Al4V with nanotubes
cp-Ti-NTs: cp-Ti with nanotubes
VEGF: vascular endothelial growth factor
NO: nitric oxide
e-NOS: endothelial nitric oxide synthase
Al_2_O_3_: alumina
*S. aureus*: *Staphylococcus aureus*
BCAECs: bovine coronary artery endothelial cells
VEGFR2: vascular endothelial growth factor receptor 2
RT: room temperature
FE-SEM: field-emission scanning electron microscopy
TEM: transmission electron microscopy
EDX: energy dispersive X-ray spectroscopy
AFM: atomic force microscopy
RMS: root mean square
SD: standard deviation
ECM: extracellular matrix
p-eNOS: phospho-endothelial nitric oxide synthase
p-VEGFR2: phospho-vascular endothelial growth factor receptor 2
MAPKs: mitogen-activated protein kinase
